# Microbial biodegradation of landfill leachates located in São Paulo state, Brazil

**DOI:** 10.1186/1753-6561-8-S4-P192

**Published:** 2014-10-01

**Authors:** Marcela Veiga, Ingrid Avanzi, Louise Hase, Marcela Baltazar, Elen Perpetuo, Roberto Guardani, Luciana Gimenes

**Affiliations:** 1 Universidade de São Paulo, SP, Brasil

## Background

Leachate is one of the most problems present in landfills, mainly due to large production and the ability to cause serious environmental damages next to these areas. An activated sludge process is commonly used for biodegrading organic contaminants in wastewater using a mixed population of microorganisms. This technology is consolidated for wastewater treatment, and recently has been used as the first step in leachate treatment process. In this work, initially, physical-chemical and biological parameters of the leachate from different landfills (Areais and Bandeirantes, both in SP), were evaluated. Also the microbial consortium present in the sludge from a wastewater plant of the Capuava Petrobras Refinery, (SP) was characterized. Purpose of this work was to study the kinetic properties of the activated sludge process by removal of the pollutant organic matter, represented by the decrease of Total Organic Carbon (TOC). In order to verify the bioremediation efficiency, experiments were developed comparing the leachates before and after biological treatments. Results show that combination of leachate and sludge can decrease the organic load significantly, demonstrating bioremediation efficiency.

## Methods

Bacterial selection was performed by their potential to degrade the leachate and were isolated and identified by growth on selective medium at 37°C. Cellular growth evaluation was performed after 24 hours of incubation and after this time were submitted to identification by mass spectrometry. Analyzed parameters of leachates were: quantification of metals by using an inductively coupled plasma optical emission spectrometry (ICP-OES), ammoniacal nitrogen, phenol, BOD and COD. Biodegradation experiments were performed in flasks incubated at 37°C in an orbital shaker (200 rpm) following the conditions: 100% (containing 120 ml of leachate and 30ml of sludge), 50% (containing 75ml of leachate, 30ml of sludge and 45ml of minimal mineral medium) and a control containing 120ml of minimal mineral medium and 30ml of sludge. Samples were taken every day for 15 days, filtered on 0.45µm, diluted 4 times in distilled water and subjected to TOC system (Thermo) to compare degradation of the leachates.

## Results and conclusions

The identification of isolated bacterial strains was performed by mass spectrometry (MALDI-TOF). This technique produces a spectrum of bacterial proteins which are analyzed by a software, comparing the profile of each database, confirming usual molecular identification. Isolated strains were identified as *Pseudomonas aeruginosa, Micrococcus luteus *and *Enterobacter cloacae*, being all them common microorganisms present in the sludge process [1]. The leachate characterization from Areais landfill showed lower concentrations of contaminants than the Bandeirantes landfill. Main parameters analyzed were: the difference in color, quantity of phenol, Chemical Oxygen Demand, Biological Oxygen Demand and presence of metals. Explanation for this may be due to its location; time of operation; composition of received wastes, among others. However, biodegradation assays showed that the isolated strains were able to degrade significantly both leachates, being an effective alternative for a primary treatment, but for this is important to keep the test conditions favorable for the growth of microorganisms [3].
